# Industrial structure upgrading, population aging and migration: An empirical study based on Chinese provincial panel data

**DOI:** 10.1371/journal.pone.0291718

**Published:** 2023-10-12

**Authors:** Dan Ma, Kai Zhou, Jingwen Xu, Yihan Qian

**Affiliations:** 1 School of Finance and Economics, Wuxi Institute of Technology, Wuxi, Jiangsu, China; 2 School of Artificial Intelligence, Nanjing Xiaozhuang University, Nanjing, Jiangsu, China; Universiti Utara Malaysia, MALAYSIA

## Abstract

Population aging and migration are two important phenomena in the process of social development in China, which have a significant impact on industrial structure upgrading. This study explores the moderating effect of the population migration on the population aging impacting the industrial structure upgrading based on Chinese provincial panel data from 2000 to 2021. The results demonstrate that the population aging has become a development trend of China’s population, and it has a significantly hinder industrial structure upgrading. Furthermore, the population migration resulting diffusion and convergence of economic factors has a meaningful moderating effects on the population aging impacting the industrial structure upgrading. Our study suggests that a reasonable and orderly population migration is critical in achieving stable and sustainable industrial structure upgrading, especially in the context of the China’s population aging.

## 1. Introduction

With the new stage of China’s economic development and the changes in the global economic environment, the government has put forward the acceleration of a “dual circulation” development pattern. This pattern focuses on the domestic economic cycle while the international economic cycle remains an extension and supplement. Specifically, it includes the development of metropolitan areas and urban agglomerations, transformation and upgrading of industrial and consumption structures, and the promotion of digital economy and green development [1, 2]. However, the practical challenge lies in the lagging development of the economic factor market, these economic factors diffusing and converging with population migration. Thus, there is a need to promote the reform of spatial planning and public resource allocation while respecting the market signals of population flow, which could greatly facilitate the effective allocation of market resources and achieve significant social impact.

Population aging is a significant issue in China has accelerated the process. Undeniably, this is the cumulative effect of several factors, such as improvements in medical care, and the change in childbearing concept [3–5]. The proportion of the elderly population aged 60 and above reached 10% in 2000, marking China’s entry into an aging society. The seventh national census in 2020 showed that the proportion of the elderly population had reached 18.7%, and this aging population structure will impact the industry development from both the supply and demand sides. The labor force’s quality and absolute quantity will decrease due to population aging, and the demand structure and subject will adjust also [6–8]. The traditional labor-intensive industry growth model has become unsustainable, and labor costs have increased rapidly, compressing the industry’s development potential [9]. Meanwhile, the “change the method and adjust the structure” approach has promoted the optimization and upgrading of industrial structure. Meanwhile, population migration, which is the main force of urban-rural dual population flow, plays a crucial role in the discussion of the relationship among population aging and industrial structure upgrading. Large-scale population movements in a vast area will undoubtedly have a significant impact on the population aging of various regions under China’s vast territory and huge population.

To enhance China’s industry’s competitiveness and sustainable growth momentum in the world, an in-depth and clear understanding of the interaction among industrial structure upgrading, population aging and migration is essential. The main contributions of this paper are as follows: (1) Incorporating population migration into the analysis framework of population aging and industrial structure upgrading, and proposing corresponding theoretical assumptions. (2) Designing two relationship models of the rationalization and advancement of industrial structure, which can effectively obtain the correlation between industrial structure upgrading and population aging and migration. (3) Based on China’s provincial panel data, empirically analyzing the impact of population aging on industrial structure upgrading, and the moderating effect of population migration on them.

The rest of the paper is organized as follows: Section 2 reviews the theoretical framework. In Section 3 gives the theoretical assumptions and analysis. In Section 4 introduces the relationship of population aging, population migration and industrial structure upgrading. In Section 5 presents the empirical results and analysis. Finally, we give the conclusions in Section 6.

## 2. Theoretical framework

The development of society and economy is inherently linked to population and industry, and the relationship between population structure and industrial structure is a commonly discussed topic. A favourable population structure can lead to the upgrading of the industrial structure, enhance productivity, and improve people’s quality of life. To gain a deeper understanding of the relationships, this section summarizes relevant literature from three perspectives, including population aging and industrial structure, population migration and industrial structure, and population migration and population aging.

Based on the available literature, the impact of population aging on industrial structure can be categorized into two groups. The first is that population aging has an inhibitory effect on the upgrading of industrial structure. Wang et al. (2015) argued that the decline in the absolute size of the labor force due to population aging, which makes difficult for the industrial structure to accommodate a large number of aging workers, and then leading to structural unemployment and hindering the upgrading of industrial structure [10]. Similarly, Long et al. (2021) found that population aging leads to changes in the age structure of the labor force, resulting in a greater labor force in the primary industry and a relatively smaller workforce in the secondary and tertiary industries, which can negatively impact labor-intensive industries [11]. Second, population aging can promote industrial structure upgrading. Cai et al. (2021) found that a decrease in the labor supply due to population aging would increase labor costs, prompting enterprises to introduce social capital and new technologies to replace the labor force, thus promoting industrial structure upgrading [12]. Additionally, Qi et al. (2020) noted that the growing proportion of the elderly population could stimulate the development of consumer service industries catering to this demographic [13]. Overall, the impact of population aging on industrial structure upgrading is still an ambiguous issue that requires further discussion and analysis.

Population is a critical carrier of material flow, information flow, capital flow, and technology flow. The migration of populations among various cities is often regarded as the spatial reconfiguration of production factors [14, 15]. Studies have shown that changes in the industrial structure will affect labor demand at different levels, which is a crucial factor in population migration among adjacent cities in modern society. The migrated population promotes economic and cultural exchanges between different regions, distributes labor resources reasonably and effectively, and narrows the development gap between these regions. Urbanization plays an important role in industrial structure upgrading and population migration, and the related issues of rural labor transfer to cities remain the focus of attention. For example, from the perspective of rural surplus labor, Teixeira et al. (2016) found that the upgrading of the industrial structure accelerates the transfer of rural surplus labor resources to urban industries and service industries, which changes the endowment of production resources in rural areas, thereby promoting the replacement of labor by social capital to promote the friendly interaction between economic development and industrial structure [16]. The orderly transfer of rural surplus labor to urban industries and services has a significant impact on the optimization of the industrial structure in two regions, reducing the difference in industrial distribution structure and inter-industry income. Additionally, the replacement of labor-intensive industries by capital-intensive industries will promote the transfer of labor and rapid economic development. Therefore, in terms of improving the efficiency of industrial structure upgrading, the population migration is an indispensable part of promoting a more abundant supply of talents, more efficient circulation of consumer goods, and faster capital accumulation.

Population migration can have an important impact on the regional labor supply and demand imbalance caused by population aging, potentially exacerbating or alleviating issues such as the number of employed individuals, industrial product consumption demand, and socio-economic vitality. Consequently, migration has become a major factor influencing regional population aging. Generally, different age groups exhibit different migration tendencies. Younger individuals, who possess high levels of education and are less constrained by asset ownership and resource allocation in their regions, are more likely to migrate. Gong et al. (2022) posit that the influx of a large mobile population in the eastern region has considerably eased the pressure of population aging. Conversely, the emigration of individuals from the central and western regions has increased the burden of population aging in those areas [17]. Zhong et al. (2019) conducted an empirical analysis using provincial panel data, which demonstrated that the ability of the eastern region to attract low-grade labor from the central and western regions weakens as the growth rate of development scale slows down [18]. Meanwhile, the rapid economic development of the central and western regions has the potential to attract more labor, which can moderate the degree of population aging in those areas. The transfer of labor from rural areas to urban has always been a research focus in China’s population, closely related to the continuous growth of the urbanization rate, which has rapidly increased from 19.39% in 1980 to 63.89% in 2021. Based on the latest census data, Liu et al. (2021) found that the proportion of urban-rural migration in China is rising, and the resident population is converging from remote rural areas to central cities, which accelerates the aging of the rural population and alleviates the aging problem in central cities to some extent [19]. Therefore, studying the moderating effect of population migration is crucial in understanding the relationship between population age and industrial structure upgrading.

Despite scholars generally examining the impact of population aging or migration on industrial structure upgrading, little attention has been paid to the interplay among all three factors. This gap leads to a lack of theoretical and empirical analysis on the moderating effect of population migration on the impact of population aging on industrial structure upgrading. To address this issue, we integrate population aging, population migration, and industrial structure upgrading within the same framework, and conduct empirical research from the perspective of industrial structure rationalization and upgrading. Our study not only examines the impact of population aging on industrial structure upgrading but also explores the moderating effect of population migration on the first two.

## 3. Theoretical assumptions and analysis

To facilitate the discussion of the interrelationship among industrial structure upgrading, population aging and migration, the following research assumptions are proposed from theoretical analysis perspective.

(1) Population aging and industrial structure upgrading.

There is a negative correlation between the population aging and the supply of working-age labors. As population aging increases in a country, it is a decrease in the working-age labor force. To maintain development, enterprises increase labor costs to obtain the required labor, which decreases their profits. To maintain profitability, enterprises may introduce new capital to upgrade the original technology and replace labor with capital in the production process. This increase in research and investment stimulates technological progress, leading to industrial structure upgrading and improved production efficiency. Based on this assumption, it can be concluded the following conclusions. Firstly, emerging industries with high production efficiency, such as modern service industry, modern manufacturing industry, and digital information industry, have intensified competition among different industries, causing a relative decline in the output efficiency of traditional labor-intensive industries. This shift eliminates industries that lag behind in technological upgrading and do not obtain sufficient cost advantage and profit space. Secondly, the imbalance between the supply and demand of the labor force, caused by population aging, forces the original labor employment structure to change. Ordinary skilled workers take the initiative to engage in education training and skill upgrading to obtain more competitive positions, thereby improving the quality of the overall labor group and the level of human capital. Finally, the demand gap generated by the industrial structure upgrading for high-skilled workers creates an imbalance in demand for ordinary labor and high-skilled labor force, leading the latter to spend more time on skills and education and training, thereby increasing the age structure of the entire social population. Based on the above analysis, the hypothesis H1 is proposed:

**Hypothesis H1: Population aging has a certain negative impact on the industrial structure upgrading**.

(2) Population migration, population aging, and industrial structure upgrading.

The transfer of population, capital, and goods is crucial to regional development, as demonstrated by the saying “to get rich before building roads.” In the process of population aging, accelerating population migration and achieving regional population stability can effectively integrate essential factors for economic development. Population migration is a typical feature of urbanization driven by differences in economic development levels and employment opportunities between rural and urban areas. This migration predominantly involves labor, resulting in economic and technological diffusion and convergence and influencing regional population aging. The different ways and quantities of population migration have a differential impact on industrial structure upgrading, leading to two contrasting outcomes for regions with net immigration and net emigration. Net immigration increases the scale of the working-age population, generating a virtuous cycle that improves the fertility rate and ensures future labor supply, thus alleviating population aging. In contrast, net emigration reduces the future working-age population and exacerbates population aging. The increase in per capita resource occupancy in regions with net emigration can lead to the scale economy effect. Based on the above analysis, we propose the hypothesis H2:

**Hypothesis H2: Population migration has significant moderating effects in the process of population aging on the upgrading of industrial structure**.

## 4. Relationship of industrial structure upgrading, population aging and migration

### 4.1 Relationship model construction

To evaluate the effectiveness of the relationship model, this paper examines the upgrading of the industrial structure from the perspectives of rationalization and advancement. The rationalization of industrial structure is measured using the Thiel index method proposed by Lu et al. (2019) [6] and Chen et al. (2020) [20]. This method was originally introduced by Thiel (1967) using the concept of entropy in information theory to calculate income inequality between individuals or regions. A higher Thiel index value indicates a greater degree of difference. The formal expression of the Thiel index is as follows:

$$\begin{array}{c} ins1=\sum_{i=1}^{3}(\frac{p_{i}}{P})\;\text{ln}\;(\frac{p_{i}\cdot L}{l_{i}\cdot P}). \end{array}$$ (1)

Where *P* represents the gross domestic product, *L* represents the total employment, *i* = 1, 2, 3 represent the primary, secondary, and tertiary, respectively. The Thiel index is denoted by *ins*1 and is used to measure the degree of inequality in the distribution of economic activities across sectors. Specifically, when *ins*1 = 0, it indicates that all industries have the same level of labor productivity and the allocation of labor across the industrial structure is reasonable. Therefore, as tends to 0, the industrial structure becomes more reasonable.

The advancement of industrial structure adopts the advanced measurement method of the primary, secondary and tertiary proposed by Fu (2010) [21] and Lu et al. (2019) [6]. As the economy continues to grow, the industrial structure undergoes regular changes, which are primarily reflected in the rising proportion of the three industries in the order of primary, secondary, and tertiary. The formal expression of the advancement of industrial structure upgrading is as follows:

$$\begin{array}{c} ins2=\sum_{k=1}^{3}\sum_{j=1}^{k}\theta_{j}. \end{array}$$ (2)

Where *k* and *j* signify the industry serial number, and their value range is [1, 2, 3], *θ*_*j*_ denotes the angle value between the added value vector of the *j*th industry and the corresponding industry value vector, whose specific formal expression is:

$$\begin{array}{c} \theta_{j}=\text{arccos}(\frac{P_{j}\cdot P_{0}}{|P_{j}\left|\cdot \right|P_{0}|}),\;\text{s.t.}\;j=1,2,3. \end{array}$$ (3)

Where $P_{0}=\left(p_{1,0}^{\prime },p_{2,0}^{\prime },p_{3,0}^{\prime }\right)$ represents a three dimensional vector composed of the proportion of the three industries’ value-added in the gross domestic product, and *P*_*j*_(*j* = 1, 2, 3) represent *P*_1_ = (1, 0, 0), *P*_2_ = (0, 1, 0) and *P*_3_ = (0, 0, 1), respectively.

Finally, in order to verify that population migration has a significant moderating effects in the process of population aging affecting industrial structure upgrading, the formal expression of the regulating effect model is constructed as follows:

$$\begin{array}{c} \left\{ \begin{array}{c} ins=\alpha_{0}+\alpha_{1}\cdot age_{i,t}+\beta \cdot X_{i,t}+\varepsilon_{i,t}\\ ins^{*}=\alpha_{0}+\alpha_{1}\cdot age_{i,t}+\alpha_{2}\cdot flow_{i,t}\cdot age_{i,t}+\beta \cdot X_{i,t}+\varepsilon_{i,t}. \end{array}\right. \end{array}$$ (4)

Where *X* represents the control variable matrix, *i* represents different provinces, *t* represents timeline, *flow* represents the value of population migration, *age* represents the value of population aging, *flow* ⋅ *age* represents the interaction term between population migration and population aging, *α* and *β* represent coefficient terms, and *ε* represents the random disturbance term.

### 4.2 Variable selection

The influence of population aging on the industrial structure upgrading and the moderating effect of population migration on the former are studied. Specifically, four comprehensive indicators are selected, including population aging, population flow, industrial structure upgrading, and control variables.

(1) Population aging is measured using the latest standard of population aging measurement adopted by the United Nations. The degree of population aging is determined by the ratio of the population aged 65 and over to the total population. The elderly dependency ratio is also an important factor in population aging.
(2) Population flow is primarily measured using two values: population migration and regional passenger traffic. Population migration is interpreted as the immigration ratio, emigration ratio, and net immigration ratio.
(3) Industrial structure upgrading is a process of industrial structure advancement. The degree of industrial structure upgrading is measured based on the rationalization and advancement of the industrial structure.
(4) Control variables are selected to alleviate the endogeneity caused by the omission of variables. Given that the population serves as the carrier of material flow, information flow, capital flow, and technology flow, five control variables are selected, including human capital, regional freight traffic, government income, education input, and patent number.

### 4.3 Data resource

To evaluate the effectiveness of our model, we selected the panel data of 31 provinces from 2000 to 2021 for empirical analysis. The data comes from the “2000–2021 China Statistical Yearbook”, “2000–2021 China Population and Employment statistical Yearbook”, and the statistical yearbook and statistical bulletins of various provinces and cities. As shown in Table 1, the econometric model employed *ins*1 and *ins*2 to represent the rationalization and advancement of the industrial structure as explained variables. The core explanatory variables were population aging (*age*), population migration (*flow*), and their interaction term (*age*_*flow*). To ensure the reliability of the conclusions, we have also selected five control variables that have a significant impact on the upgrading of the industrial structure, namely, education level (*edule*), freight transfer traffic (*tran*), financial revenue (*rev*), education inputs (*eduin*), and patent number (*pat*).

**Table 1:** Variables descriptive information.

No.	Variables	Codes	Description
1	Rationalization	*ins*1	Refer to the Thiel index method from Chen et al. (2020) and Lu et al. (2021)
2	Advancement	*ins*2	Refer to the industrial structure advancement method from Fu et al. (2010)
3	Population aging	*age*	Ratio of population aged 65 and over to total population, elderly dependency
4	Population migration	*flow*	The variables involved include immigration ratio and emigration ratio
5	Education level	*edule*	Ratio of the population above college level to the working population
6	Freight transfer traffic	*tran*	The freight traffic transported by various means of transportation
7	Financial revenue	*rev*	Income from participation of state finance in distribution of social products
8	Education inputs	*eduin*	Refer to the numbers of colleges and universities, undergraduate students
9	Patent number	*pat*	Number of patent applications and patent approvals

## 5. Empirical results and analysis

### 5.1 Data preparation

Table 2 presents the statistics information of Chinese provincial panel data, which were analyzed for the selected variables. Due to some missing values in the collected data and the tendency for variable values in the provincial panel data to show continuous linear changes, we used linear interpolation to fill in the missing values. Additionally, we employed a 1% tail reduction process for the outliers and missing values, resulting in a total of 673 balanced panel data. The first explained variable, *ins*1, represents the industrial structure rationalization index, with a mean of 0.642, a standard deviation of 0.411, a minimum value of -0.282, and a maximum value of 2.177. The second explained variable, *ins*2, represents the industrial structure advancement index, with a mean of 1.196, a standard deviation of 0.624, a minimum of 0.518, and a maximum of 5.297. The gap between *ins*1 and *ins*2 in the sample data is small and shows a left-skewed distribution. The core explanatory variable, *age*, represents the composite value of the proportion of the population aged 65 and over and the elderly dependency ratio. Its mean value is 11.290, the standard deviation is 3.135, the minimum value is 4.270, and the maximum value is 23.577. The moderating variable, *flow*, includes population immigration ratio, population emigration ratio, net immigration ratio, and passenger traffic as the third-level indicators, calculated using the entropy method. Its mean is 0.234, and the standard deviation is 0.125.

**Table 2:** The statistics information of Chinese provincial panel data.

No.	Codes	Mean	Sta.	Min	Max
1	*ins*1	0.642	0.411	-0.282	2.177
2	*ins*2	1.196	0.624	0.518	5.297
3	*age*	11.290	3.135	4.270	23.577
4	*flow*	0.234	0.125	0.115	0.781
5	*edule*	11.010	7.127	1.059	50.486
6	*tran*	11.067	1.213	4.875	12.981
7	*rev*	10.462	3.810	3.404	17.315
8	*eduin*	15.136	1.166	11.251	17.835
9	*pat*	9.046	2.032	1.946	13.701

**Fig 1:**
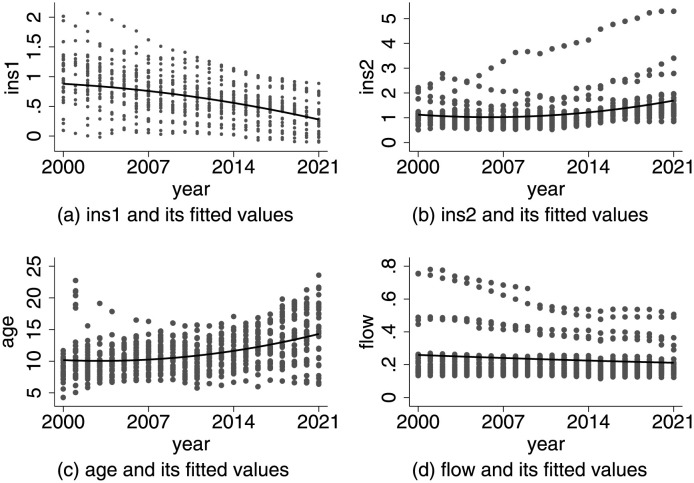
The scatter and quadratic fit of Chinese provincial panel data. (a) ins1 and its fitted values, (b) ins2 and its fitted values, (c) age and its fitted values, (d) flow and its fitted values.

Fig 1 presents the scatter plots and quadratic fits of the provincial panel data, where each dot represents the corresponding values of different provinces in different years, and each curve represents its corresponding quadratic fitting curve. In Fig 1(a), the quadratic fit curve of the rationalization of industrial structure demonstrates a decreasing trend, indicating a gradual improvement in the fitting value of the rationalization of industrial structure over time. Fig 1(b) shows an increasing trend in the quadratic fit curve of the advancement of industrial structure, suggesting an enhancement in the advancement of industrial structure. Fig 1(c) and 1(d) respectively depict the scatter and quadratic fitting trends of population aging and population flow, indicating the deepening of population aging and the changing pattern of population flow.

### 5.2 The impact of population aging on industrial structure upgrading

#### (1) Stability test

The stability test is an important step in panel data analysis. Unit Root Test and Cointegration Test are used to verify whether there is a pseudo regression, where the unit root test is used to determine if there is a unit root in the sequence. If the panel data remains stable after *d*(*d* > 1) order difference, it indicates a d-order single integer sequence, and satisfies the premise of cointegration test. This ensures that the regression analysis is not affected by non-stationary time series. Table 3 presents the results of Fisher’s unit root test on Chinese provincial panel data. When using 0-order variables for verification, the *P* values of the unit root test of all variables are close to zero and reject the original assumption of unit roots. Moreover, all continuous variables are of the same order single integer, allowing for the original panel data to be used for regression.

**Table 3:** Fisher’s unit root test of provincial panel data.

Codes	Inverse chi-squared (62)P	Inverse normal Z	Inverse logit t(159)L*	Modified inv. *χ*^2^ Pm
ins1	438.337 (0.000)	-16.613 (0.000)	-21.733 (0.000)	33.796 (0.000)
ins2	147.189 (0.000)	-5.727 (0.000)	-5.901 (0.000)	7.650 (0.000)
age	275.076 (0.000)	-11.391 (0.000)	-13.314 (0.000)	19.135 (0.000)
edule	243.123 (0.000)	-10.969 (0.000)	-11.894 (0.000)	16.265 (0.000)
tran	139.732 (0.000)	-6.491 (0.000)	-6.317 (0.000)	6.981 (0.000)
rev	201.278 (0.000)	-9.514 (0.000)	-9.803 (0.000)	12.508 (0.000)
eduin	124.820 (0.000)	-5.272 (0.000)	-5.199 (0.000)	5.641 (0.000)
pat	251.469 (0.000)	-10.888 (0.000)	-12.240 (0.000)	17.015 (0.000)

#### (2) Correlation test

The strong correlation between explanatory variables may lead to false conclusions. Hence, Pearson correlation analysis is used to exclude collinearity between explanatory variables in panel data. Table 4 shows the Pearson correlation analysis of the panel data, including the correlation coefficient between variables and its significance level. Generally, the correlation coefficient between variables less than 0.8 indicates no strong correlation. Table 4 shows that the regression coefficients between all explanatory variables are less than 0.8, indicating no strong correlation between panel data variables.

**Table 4:** Pearson correlation analysis of the panel data.

	**ins1**	**ins2**	**age**	**edule**	**tran**	**income**	**eduin**	**pat**
ins1	1.000							
ins2	-0.194(0.000)	1.000						
age	-0.665(0.000)	-0.025(0.522)	1.000					
edu	-0.613(0.000)	0.666(0.000)	0.331(0.000)	1.000				
tran	-0.465(0.000)	-0.362(0.000)	0.468(0.000)	0.152(0.000)	1.000			
income	0.197(0.000)	-0.201(0.000)	-0.138(0.000)	-0.301(0.000)	-0.118(0.002)	1.000		
inedu	-0.593(0.000)	0.030(0.0435)	0.500(0.000)	0.445(0.000)	0.792(0.000)	-0.441(0.000)	1.000	
pat	-0.756(0.000)	0.114(0.003)	0.617(0.000)	0.566(0.000)	0.750(0.000)	-0.271(0.000)	0.790(0.000)	1.000

Table 5 represents the multicollinearity test of the panel data, where VIF represents the variance inflation factor of all variables. VIF less than 10 indicates no multicollinearity in the sample data. Table 5 shows that the variance expansion factors of all variables are less than or equal to 8.740, and the overall variance expansion factor is only 4.430. Therefore, the panel data in this empirical analysis can be deemed free of multicollinearity.

**Table 5:** Multi-collinearity test of the panel data.

	age	edule	tran	rev	eduin	pat	mean
VIF	1.660	2.000	3.990	1.650	8.740	8.520	4.430
1/VIF	0.601	0.501	0.251	0.608	0.114	0.117	

#### (3) Benchmark regression

Table 6 presents the benchmark regression of population aging to the rationalization of industrial structure. The regression method gradually adds control variables. It can be seen from R-Square that the model fitting effect is good, and the regression coefficient of the core explanatory variable is significantly negative at the 1% significance level. Further analysis shows that population aging inhibits industrial structure rationalization. As the degree of aging deepens and the proportion of aging population in society increases, the rationalization of industrial structure tends to stabilize.

**Table 6:** Pearson correlation analysis of the panel data.

Codes	(1) ins1	(2) ins1	(3) ins1	(4) ins1	(5) ins1	(6) ins1
age	-0.0405***	-0.0393***	-0.0375***	-0.0375***	-0.0370***	-0.0257***
	(-12.735)	(-12.565)	(-11.863)	(-11.861)	(-12.068)	(-7.635)
edule		0.0155***	0.0123***	0.0127***	0.0054	0.0093***
		(5.032)	(3.764)	(3.861)	(1.591)	(2.792)
tran			-0.0672***	-0.0551**	-0.0023	0.0220
			(-2.909)	(-2.069)	(-0.086)	(0.833)
rev				-0.0393	0.1336***	0.1320***
				(-0.905)	(2.668)	(2.737)
eduin					-0.3543***	-0.3568***
					(-6.375)	(-6.665)
pat						-0.0779***
						(-7.008)
Constant	1.3510***	1.2765***	1.9660***	2.3955***	4.2521***	4.5091***
	(34.702)	(31.163)	(8.174)	(4.502)	(7.179)	(7.888)
Individual fixed effect	Control	Control	Control	Control	Control	Control
Year fixed effect	Control	Control	Control	Control	Control	Control
Observations	673	673	673	673	673	673
R-squared	**0.686**	**0.698**	**0.702**	**0.703**	**0.721**	**0.742**
Number of id	31	31	31	31	31	31
Ajusted R2	0.718	0.718	0.718	0.718	0.718	0.718

Remarks: t-statistics in brackets,

*, ** and *** are significant at 10%, 5% and 1%, respectively.

Similarly, Table 7 shows the benchmark regression of population aging to the advancement of industrial structure. The model fitting effect is good, and the regression coefficient of the core explanatory variables is significantly negative at the 1% significance level. The results show that population aging inhibits industrial structure advancement. As the degree of aging deepens, and the proportion of social aging population increases, the population aging inhibits industrial structure advancement.

**Table 7:** Pearson correlation analysis of the panel data.

Codes	ins2	ins2	ins2	ins2	ins2	ins2
age	-0.0346***	-0.0301***	-0.0171***	-0.0171***	-0.0168***	-0.0235***
	(-6.598)	(-6.426)	(-4.394)	(-4.438)	(-4.387)	(-5.439)
edule		0.0590***	0.0348***	0.0372***	0.0326***	0.0303***
		(12.813)	(8.724)	(9.285)	(7.721)	(7.133)
tran			-0.5012***	-0.4424***	-0.4094***	-0.4239***
			(-17.691)	(-13.668)	(-12.136)	(-12.551)
rev				-0.1921***	-0.0842	-0.0832
				(-3.641)	(-1.351)	(-1.347)
eduin					-0.2212***	-0.2197***
					(-3.199)	(-3.202)
pat						0.0463***
						(3.251)
Constant	1.3904***	1.1065***	6.2514***	8.3507***	9.5094***	9.3566***
	(21.659)	(18.063)	(21.186)	(12.920)	(12.907)	(12.772)
Individual fixed effect	Control	Control	Control	Control	Control	Control
Year fixed effect	Control	Control	Control	Control	Control	Control
Observations	673	673	673	673	673	673
R-squared	**0.434**	**0.553**	**0.703**	**0.710**	**0.714**	**0.719**
Number of id	31	31	31	31	31	31
Ajusted R2	0.693	0.693	0.693	0.693	0.693	0.693

#### (4) Robustness test

To ensure the robustness of the conclusions, the Propensity Score Matching (PSM) method was utilized for regression analysis. The specific implementation involved dividing the sample data into treatment and control groups using the Treat function, followed by observing the average treatment effect of the two groups. The *k*-nearest neighbor Caliper radius matching method was employed, with *k* set at 5 and Caliper at 0.01. This meant that among five individuals whose propensity scores differ by 1%, the closest individual was selected for 1 to 5 matching. The sample data were grouped based on the core explanatory variable of population aging, where the aging level greater than the mean value was the treatment group, and vice versa for the control group. The PSM calculation formula is as follows:

$$\begin{array}{c} \begin{array}{c} PSM=P[Treat=1|X]\\ =E[Treat=0|X]. \end{array} \end{array}$$ (5)

Where *P*[] represents the probability of being in the treatment group under observable characteristic variables, and *X* represents observable variables, also known as covariates.

Furthermore, the impact of population aging on industrial structure was studied from the perspectives of rationalization and advancement, with two explained variables, *ins*1 and *ins*2. Control variables were used as covariates to make the two groups of samples as similar as possible except for the aging level, which helped eliminate endogenous problems and estimation bias. The formula of average treatment effect (ATT) is as follows:

$$\begin{array}{c} \begin{array}{c} \text{ATT}=E[ins^{\prime }|Treat=1]\\ \;=E[ins^{\prime \prime }|Treat=1]\\ \;\;\;=E[ins^{\prime }-ins^{\prime \prime }|Treat=1]. \end{array} \end{array}$$ (6)

**Fig 2:**
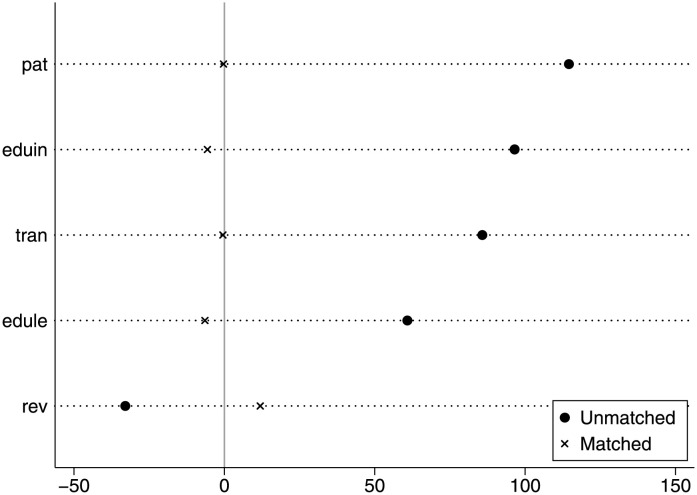
Standardized bias of covariates.

Taking the rationalization of industrial structure as an example, *ins*′ in Eq (6) represents the industrial structure rationalization of the treatment group, and *ins*″ represents the industrial structure rationalization of the control group. Fig 2 shows the standardized bias of covariates. It can be seen that the standardized bias of most variables are reduced after matching, which indicates that the matching is effective. Thus, the treatment group and the control group are more similar after matching.

**Fig 3:**
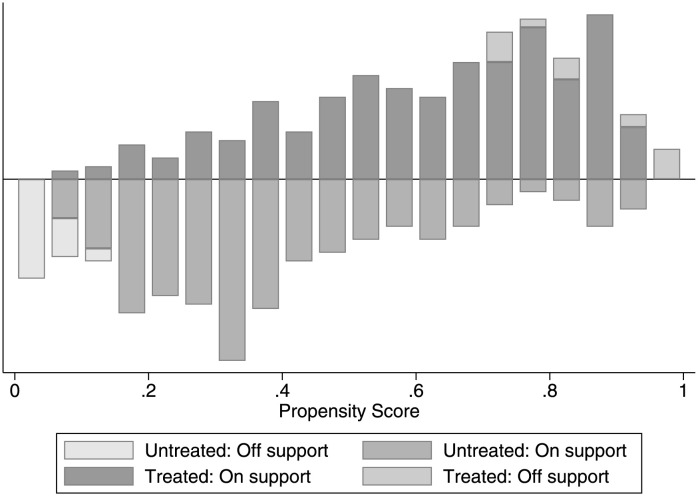
Common value range of propensity score.

Table 8 shows the standardized deviation before and after the matching of population aging to the industrial structure upgrading. The standardized bias of all variables after matching is less than 10%, and the standardized deviation of the edule variable decreases from 114.500% to -0.300%. Almost all the t-test results did not reject the original assumption that there was no systematic difference between the treatment group and the control group. Fig 3 represents the common range of propensity scores. Most of the observed values were within the common range. Although a small number of samples were lost during propensity score matching, they remained reliable.

**Table 8:** Standardization deviation before and after the matching of the population aging on the industrial structure rationalization.

Codes		Treated mean	Control mean	%bias	%reduct bias	*t*	*p* > *t*	V(T)/V(C)
edule	Unmatched	13.090	8.938	60.800	7.890	0.000	1.68*	
	Matched	12.663	13.104	-6.500	89.400	-0.720	0.474	0.79*
tran	Unmatched	11.546	10.590	85.800	11.120	0.000	0.33*	
	Matched	11.524	11.529	-0.500	99.400	-0.080	0.933	0.980
rev	Unmatched	9.842	11.081	-32.900	-4.270	0.000	0.840	
	Matched	9.930	9.481	11.900	63.800	1.600	0.110	1.240
eduin	Unmatched	15.644	14.630	96.500	12.510	0.000	0.54*	
	Matched	15.615	15.674	-5.600	94.200	-0.840	0.403	0.950
pat	Unmatched	10.058	8.037	114.500	14.860	0.000	0.63*	
	Matched	9.930	9.935	-0.300	99.800	-0.040	0.969	0.810

Table 9 shows the PSM robustness test of population aging on the industrial structure rationalization. Taking the average treatment effect represented by ATT as an example, the difference value of the *ins*1 model is -0.173, and *T* − *stat*. is -3.860. The results indicated that the above empirical analysis is strongly significant at 1% significance, and the difference between the treatment group and the control group noted that population aging could inhibit the industrial structure rationalization. Similarly, population aging also has a certain inhibitory impact on the industrial structure advancement.

**Table 9:** PSM robustness test of population aging on the industrial structure rationalization.

Codes	Sample	Treated	Controls	Difference	S.E.	T-stat.
Industrial structure rationalization	Unmatched	0.410	0.873	-0.463	0.026	-17.660
	ATT	0.428	0.602	**-0.173**	0.045	**-3.860**
	ATU	0.831	0.566	-0.265		
	ATE		-0.218			
industrial structure advancement	Unmatched	1.176	1.217	-0.042	0.048	-0.870
	ATT	1.151	1.289	**-0.137**	0.077	**-1.790**
	ATU	1.160	1.205	0.045		
	ATE			-0.048		

### 5.3 The moderating effects of population migration

#### (1) Interactive regression test

The previous analysis has shown that population aging has a negative impact on the rationalization and development of industrial structures. This section aims to study the moderating effects of population migration on the population aging impacting the industrial structure upgrading from the perspective of population flow. Table 10 presents the interactive regression test of the moderating effects of population migration on the population aging impacting the industrial structure upgrading. Column *ins*1 shows that the regression coefficient of age is still significantly negative at the 1% significance level, indicating that population aging can inhibit the rationalization of industrial structure even after considering the factor of population migration. Moreover, *age*_*flow* represents the interaction between population aging and population migration, and its coefficient is significantly positive at the 5% significance level. Therefore, population migration has a positive moderating effect on the impact of population aging on the rationalization of industrial structure. Similarly, column *ins*2 also denotes that population migration has a positive role in the process of population aging impacting industrial structure upgrading.

**Table 10:** Interactive regression test of the moderating effects of the population migration on the population aging impacting the industrial structure upgrading.

Codes	ins1	ins2
age	-0.0415*** (-7.193)	-0.0188*** (-2.722)
flow	-1.5364*** (-4.152)	-3.6286*** (-8.184)
age_flow	0.0787*** (3.883)	0.0704*** (2.902)
edule	0.0048 (1.366)	0.0162*** (3.880)
tran	0.0234 (0.892)	-0.4090*** (-12.981)
rev	0.1096** (2.277)	-0.1470** (-2.547)
eduin	0.3404*** (-6.227)	-0.0960 (-1.465)
pat	-0.0776*** (-6.747)	0.0150 (1.089)
Constant	4.9513*** (8.516)	9.4280*** (13.534)
Individual fixed effect	Control	Control
Year fixed effect	Control	Control
Observations	673	673
R-squared	0.749	0.764
Number of id	31	31
Ajusted R2	0.741	0.741

#### (2) Grouping regression test

To further verify the moderating effects of population migration, a grouping regression test method is employed on the moderating effects of population migration on industrial structure upgrading. The samples are divided into two groups based on the mean value of the variable population mobility. The high-value group of population migration is larger than the mean value, and the low-value group of population migration is less than or equal to the mean value. Table 11 presents the test results for these groups. Taking the explained variable *ins*1 as an example, the regression coefficient of age for *ins*1 (2) is significantly negative at the 1% significance level, and the regression coefficient is considerably larger than the *ins*1 (1). Additionally, we introduce an uncorrelated model test method to determine if there are significant differences between the two sets of regressions. When the test passes, it indicates that the coefficients of the two groups of regressions can be compared. From Table 11, the test coefficient is significantly 8.18 at the 1% significance level, indicating that the regression coefficients of age in regression (1) and regression (2) can be compared. That is, population migration has a significant moderating effects on the impact of population aging on the rationalization of industrial structure. Similarly, the results of regression (3) and regression (4) also show the same trend, i.e., population migration has a significant moderating effects on the population aging impacting the industrial structure advancement.

**Table 11:** Group regression test of the moderating effects of the population migration on the population aging impacting the industrial structure upgrading.

Codes	(1) ins1	(2) ins1	(3) ins2	(4) ins2
age	high-value	low-value	high-value	low-value
	-0.0150** (-2.403)	-0.0413*** (-9.273)	-0.0064 (-0.858)	-0.0289*** (-5.949)
edule	0.0049 (1.010)	0.0040 (0.841)	0.0393*** (6.874)	-0.0080 (-1.519)
tran	0.0055 (0.132)	0.0233 (0.634)	-0.6026*** (-12.064)	-0.1020** (-2.548)
rev	0.0124 (0.169)	0.2899*** (4.545)	-0.2152** (-2.467)	-0.3862*** (-5.566)
eduin	-0.4206*** (-4.723)	-0.2798*** (-4.413)	0.0890 (0.840)	-0.0819 (-1.187)
pat	-0.0580*** (-3.095)	-0.0974*** (-6.385)	-0.0068 (-0.307)	0.0286* (1.727)
Constant	6.9211*** (7.948)	1.5842** (2.196)	8.9617*** (8.645)	8.6851*** (11.064)
Group test	8.18***		3.97**	
Individual fixed effect	Control	Control	Control	Control
Year fixed effect	Control	Control	Control	Control
Observations	336	337	336	337
R-squared	0.713	0.844	0.816	0.760
Number of id	18	20	18	20
Ajusted R2	0.722	0.722	0.722	0.722

In summary, population migration, as a carrier of material flow, information flow, capital flow, and technology flow, has significant moderating effects on the impact of population aging on industrial structure upgrading. This is mainly manifested in two aspects. Firstly, it can significantly alleviate the degree of population aging in the immigration areas, and the industrial structure will also achieve benign rationalization and advancement. Secondly, it can significantly deepen the degree of population aging in the net emigrate areas, and the original industrial structure will also undergo destruction and reconstruction, eventually returning to the rationalization and development process at some point in the future.

## 6. Conclusions

In this paper, we have proposed theoretical assumptions about the interrelationships among industrial structure upgrading, population aging and migration. Two relationship models of the rationalization and advancement of industrial structure were designed to demonstrate the proposed assumptions. The results indicate that the deepening of population aging has an unprecedented negative impact on the upgrading of industrial structure, while the convergence of economic factors brought by population migration has a positive impact on the upgrading of industrial structure.

Based on the above works, the following conclusions are drawn. First, there is a need to fully explore the resources of the elderly group and optimize industrial structure upgrading. As the rule of population development, the emergence of aging is an irreversible trend. A rational layout is required to leverage the catalytic effect of population aging and utilized to promote the upgrading of industrial structure. Second, orderly population migration should be guided, and a precise match between population migration and industrial structure upgrading should be established. The development of capital productivity inevitably expands the production and social division of labor, and population mobility plays an important role in it. Policy guidance and training and education can make the floating population better adapt to the upgrading of industrial structure in the inflow areas. Third, starting from the top-level design of the nation, coordinated development of population structure, population mobility, and industrial structure should be pursued. Industries should be rationally distributed in different regions to create a stable and sustainable driving force for the upgrading of industrial structure in various regions.

## Supporting information

S1 Data (XLS)

## References

[1] Feng C, Xia YS, Sun LX. Structural and social-economic determinants of China’s transport low-carbon development under the background of aging and industrial migration. Environmental Research, 2020, 188: 109701. doi: 10.1016/j.envres.2020.109701 32504850

[2] Zhou SD, Han JQ, Ge JH, Sheng MW. Theoretical Exploration of Domestic and International Dual Circulation Strategy with Domestic Grand Circulation Being the Mainstay. Journal of Nanjing Agricultural University (Social Sciences Edition), 2021, 21(3): 22–29.

[3] Peng XZ. Coping with population ageing in mainland China. Asian Population Studies, 2021, 17(1): 1–6. doi: 10.1080/17441730.2020.1834197

[4] Yu HX, Lu LF. Research on the Relationship between Aging, Population Mobility and Economic Growth. Price Theory and Practice, 2021, 9: 82–85.

[5] Chen X, Zheng YL, Yao D. Population aging, industrial intelligence and high-quality economic development. Statistics and Decision, 2022, 6: 129–132.

[6] Lu J, Wang XF, Liu L, Chen Y. Synergy Effects of Aging, Population Migration and Industrial Structure in China. Economic Geography, 2019, 39(9): 39–47.

[7] Fan M, Yang P. Research on the relationship between population Aging, Digital Finance and Industrial structure upgrading. Price Theory and Practice, 2021, 4: 108–111.

[8] Zou JX, Ji RD, Mao R. The two-way interaction between population aging and industrial transformation. Economics of Transition and Institutional Change, 2022, 30(2): 311–335. doi: 10.1111/ecot.12295

[9] Hosan S, Karmaker SC, Rahman MM, Chapman AJ, Saha BB. Dynamic links among the demographic dividend, digitalization, energy intensity and sustainable economic growth: Empirical evidence from emerging economies. Journal of Cleaner Production, 2022, 330: 129858. doi: 10.1016/j.jclepro.2021.129858

[10] Wang W, Liu YF, Peng DD. Research on Effects of Population Aging on Industrial Upgrading. China Industrial Economics, 2015, 11: 47–61.

[11] Long HM, Yan WZ, Yang JJ. The Impact of Population Aging on the Upgrading of Industrial Structure: Promotion or Suppression?——Empirical Analysis Based on the Perspective of Financial Structure. The Theory and Practice of Finance and Economics, 2021, 42(6): 44–51.

[12] Cai X. Population Aging, Urbanization and Technological Innovation. Statistics and Decision, 2021, 37(23): 76–80.

[13] Qi HQ, Liu Y. Change of population age structure and the upgrading of household consumption: an empirical research based on CFPS data. China Population, Resources and Environment, 2020, 30(12): 174–184.

[14] Li TZ, Lu MJ. Research on the Characteristics and Influencing Factors of China’s Population Flow Network——Based on Tencent Location Big Data Analysis. Contemporary Economic Management, 2022, 44(2): 1–9.

[15] Qin LL, Lin RF, Gao RW, Lin H. Correlation between Population Structure and Regional Innovation Ability Based on Big Data Analysis. Mathematical Problems in Engineering, 2022: 1–10. doi: 10.1155/2022/5250853

[16] Teixeira AA, Queiros AS. Economic growth, human capital and structural change: A dynamic panel data analysis. Research policy, 2016, 45(8): 1636–1648. doi: 10.1016/j.respol.2016.04.006

[17] Gong F, Deng LZ. Population Aging, Cross-District Population Mobility and County Economic Growth. Journal of Zhongnan University of Economics and Law, 2022, 1: 147–160.

[18] Zhong R. Spatial Transfer, and Planning Strategies of Urban-Rural Aging Population Reversed in China: From the Prospective of Population Migration. Urban Development Studies, 2019, 26(2): 24–30.

[19] Liu JH, Liu Q. The Characteristics, Influence and Coping Strategies of the New Form of Aging Society in China: Interpretation Based on the Data of the Seventh Census. Population and Economics, 2021, 5: 13–24.

[20] Chen J. Population Aging, Industrial Structure Adjustment and Economic Growth: An Empirical Analysis Based on Jiangsu. East China Economic Management, 2020, 34(2): 18–23.

[21] Fu LH. An Empirical Research on Industry Structure and Economic Growth. Statistical Research, 2010, 27(8): 79–81.

